# The effect of flash vacuum cooling on the flavor of ultrapasteurized milk

**DOI:** 10.3168/jdsc.2022-0215

**Published:** 2022-03-31

**Authors:** B.G. Carter, Y. Jo, D.C. Cadwallader, MaryAnne Drake

**Affiliations:** Department of Food, Bioprocessing and Nutrition Sciences, Southeast Dairy Foods Research Center, North Carolina State University, Raleigh 27695

## Abstract

Ultrapasteurization (UP) extends the shelf life of milk. Direct steam injection (DSI) is commonly used for UP because milk is quickly heated and cooled. During this process, steam is directly injected into milk and removed by a vacuum cooler. Consumers do not prefer the flavor of DSI-UP milk compared with traditional high temperature short time (HTST) milk due to the higher cooked and eggy flavors of DSI-UP milk. The objective of this research was to characterize the effect of the vacuum cooler on the flavor of DSI-UP milk. Raw skim milk was pasteurized at 140°C for 2.3 s by DSI and homogenized at 20.7 MPa. By using a liquid sample port, steam-infused pasteurized milk was sampled after heating but before reaching the vacuum chamber. A septum was installed in the vacuum chamber to allow sampling of the removed volatiles by solid-phase microextraction (SPME) fiber followed by gas chromatography-triple quadrupole mass spectrometry (GC-MS/MS) combined with a sulfur-selective flame photometric detector. Steam-infused milk and vacuum-cooled milk diluted to the same solids contents were evaluated by descriptive sensory analysis and volatile compound analysis. The entire experiment was replicated 3 times. Milks cooled by the vacuum cooler were lower in sweet aromatic, sulfur/eggy, and cooked flavors than milk sampled before the vacuum cooler. Volatile compounds removed by the vacuum cooler included the sweet aromatic flavor contributors furaneol, maltol, furfural, sotolon, 2-heptanone, γ-dodecalactone, γ-decalactone, and δ-decalactone, as well as the cooked and sulfur/eggy contributors hydrogen sulfide and dimethyl sulfide. The vacuum cooler applied during DSI-UP of milk is effective at removing steam and cooling UP milk, but this process may also remove important flavor compounds from fluid milk.

## Highlights

• Steam-infused milk and vacuum-cooled milk at the same solid contents had distinct flavors.
• Vacuum-cooled milks had decreased sweet aromatic and sulfur/eggy flavors by descriptive analysis.
• Aroma-active compounds were removed from milks by the vacuum cooler, as measured by GColfactometry and GC-MS.
• Vacuum cooling of steam-infused milk removed sweet aromatic and milky volatiles and sulfur volatile compounds.

## Graphical Abstract

**Figure:**
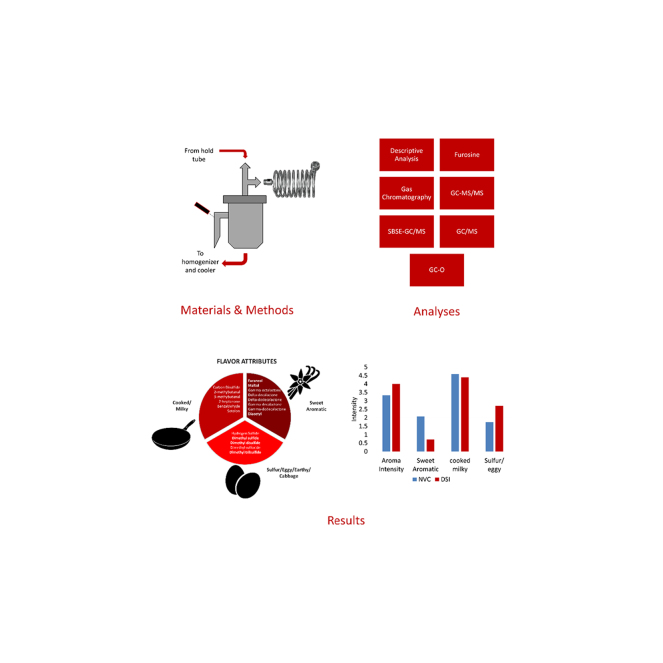
**Summary:** This study characterized the effect of the vacuum cooler on the flavor of ultrapasteurized (UP) milk. Raw skim milk was pasteurized at 140°C for 2.3 s by direct steam injection (DSI) and homogenized. Steam-infused pasteurized milk was sampled after heating but before it reached the vacuum chamber with the use of a liquid sample port. Steam-infused milk and vacuum-cooled milk diluted to the same solids content were evaluated by descriptive sensory analysis and volatile compound analysis. Milks cooled by the vacuum cooler were lower in sweet aromatic, sulfur/eggy, and cooked flavors than milk sampled before the vacuum cooler (*P* < 0.05). Volatile compounds removed by the vacuum cooler included sweet aromatic flavor contributors as well as cooked and sulfur/eggy flavor-contributing volatile compounds. The vacuum cooler applied during DSI of UP milk is effective at removing steam and cooling UP milk, but this process may also remove important flavor compounds from fluid milk

The dairy industry has been shifting toward extended shelf life milk. Traditional HTST-pasteurized (72°C for 15 s) milk has a relatively short refrigerated shelf life of 2 to 3 wk. This ~18-d refrigerated shelf life puts a strain on the dairy industry to distribute product quickly and get it into the hands of the consumer. Ultrapasteurized (**UP**) milk uses a high-heat pasteurization process where milk is heated and held at 138°C for at least 2 s. The higher heat treatment of UP milk extends the refrigerated shelf life to 60 to 70 d, and these milks are called extended shelf life milks. Shelf life can be extended by using UP; however, adverse flavor effects can occur (Lee et al., 2017).

Two main methods of UP are used in the dairy industry. Indirect (**IND**)-UP involves the indirect heating of the milk by either a plate, tube-in-tube, or tube-in-shell heat exchanger. This process requires a lot of time to warm and reach process temperature, as well as time to cool for storage, which leads to greater overall heat load on the milk (Lee et al., 2017). The second method is direct steam injection (**DSI**); DSI milk has steam injected directly into the milk to heat it rapidly, limiting the heat load on the milk with the same level of lethality. The water added by the steam is later removed by a vacuum chamber, which also serves to cool the product (Datta et al., 2002). In recent years, DSI has become more popular because of the efficient heat transfer as well as the lower total heat exposure, which leads to a higher quality product.

These 2 heating methods for UP produce different flavor profiles in UP milk. Lee et al. (2017) demonstrated that DSI milk was lower in sweet aromatic flavor and higher in sulfur/eggy flavor than IND-UP milk (Lee et al., 2017). Both UP milks had higher cooked flavor and distinct sulfur flavor than HTST milk, regardless of fat content. The differences in the flavor profile of IND and DSI milk suggest that the vacuum chamber may play a key role in the flavor differences of these 2 milks.

During DSI-UP production, a vacuum chamber is used to remove excess water added by the steam and to quickly cool the milk. Vacuum is often used in the food industry because it lowers the boiling point of water, allowing evaporation of water at lower temperatures. The vacuum pressure also affects the boiling point and vapor pressure of volatile flavor compounds. Under vacuum, these compounds more easily volatilize into the headspace, which makes them more susceptible to removal by the vacuum pump. This effect has been noted before in the vapor separator of an evaporator for the production of milk powder (Park and Drake, 2016). Milk concentrates produced without vacuum evaporation to the same solids and heat load were higher in typical milky, sweet aromatic flavors and volatile compounds than concentrates produced with vacuum evaporation. Analysis of the vapor removed from the milk revealed that many milky and sweet aromatic compounds were removed by the vacuum.

Even though the use of vacuum gives the industry better processing control, it may remove a certain fraction of important flavor components. This problem may be exaggerated in flavored milks and protein beverages. Flavors are often the most expensive ingredients included in beverage formulations and may be the most affected by vacuum cooling. Milk processed by DSI-UP already has intense cooked and sulfur flavors due to the extreme heat treatment, and the vacuum chamber may be selectively removing desirable flavor compounds (sweet aromatic) and intensifying undesirable flavors (sulfur). The purpose of this research was to determine the effects of the vacuum cooler on the flavor of DSI-UP skim milk.

For this study, on 3 different occasions, raw skim milk (3.4% protein, 0.08% fat, coliforms <10^1^ cfu/mL, aerobic plate count <10^2^ cfu/mL, SCC <50,000 cells/mL) was obtained from the North Carolina State University Dairy Education System. An EHVH pasteurization unit (Microthermics) with a 2-stage homogenizer (model NS2006H, GEA Niro Soavi) was used to ultrapasteurize the milk. Before processing the milk, the system was sterilized by heating the unit to 121.1°C recorded by the last thermocouple and held at that temperature for 20 min. Raw skim milk was processed at a flow rate of 1.4 L/min. The milks were preheated to 90°C, heated to 140°C for 2.3 s under 330 kPa pressure by direct culinary steam injection (model LG30, Electro-Steam Generator Corp.) using a Microthermics Steam Injection Module with Cub 5 software (version 3.1; Microthermics), and then cooled to 85°C under a vacuum of 1,040 mmHg to remove both heat and added water. The DSI-UP milk was then homogenized (20.7 MPa total, 3.4 MPa second stage), cooled to 10°C, collected into glass jars (500 mL, VWR), cooled to 4°C in an ice bath, and stored at 4°C in the dark until analysis. Analyses were conducted within 8 h. Hold times were calculated under laminar flow assumptions.

To isolate the direct effect of the vacuum chamber but maintain the same heating profile, modifications were made to the DSI-UP system (Microthermics) to sample milk after the hold tube and before the vacuum chamber. Milk was diverted through a cooling coil placed into an ice bucket to produce milk that was not vacuum cooled (**NVC**) but that was cooled indirectly and placed into glass jars. This method did not remove the added water caused by steam injection. Both DSI and NVC milks were diluted to 8.6% solids (wt/vol) with deionized water as determined by a CEM microwave moisture analyzer (SMART 5, CEM Corp.) and a calibrated mid-Fourier-transform infrared analyzer (Lactoscope FTA, Delta Instruments).

To better understand flavor compounds removed directly by the vacuum chamber, a sampling septum (Fisher Scientific) was installed at the entrance of the vacuum pump to allow sampling of the vapor. Before collection of fluid milk samples, 9 solid-phase microextraction fibers [2 cm divinylbenzene/carboxen/polydimethylsiloxane (DVB/CAR/PDMS) fiber; Supelco] were exposed for 2 min through a silicone septum (Fisher Scientific) in the exhaust of the vacuum chamber to collect qualitative information about which flavor compounds were removed in the vapor. The entire experiment was repeated in triplicate.

Furosine (**FUR**) was measured as described by Resmini et al. (1990) with modifications. Two milliliters of milk was dissolved in 6 mL of 10.6 *M* HCl (Sigma Aldrich) into a glass tube. Nitrogen gas was bubbled into the tube for 2 min followed by sealing glass tubes with Teflon-lined lids. The samples were hydrolyzed at 110°C for 24 h and then cooled to room temperature. Next, 100 µL of hydrolysate was added to 900 µL of HPLC-grade water (Fisher Scientific). The diluted hydrolysate was centrifuged at 14,000 × *g* for 10 min, and the supernatant was placed in HPLC autosampler vials with Teflon-lined lids (Waters Corp.).

Separation and detection of FUR was done by reversed-phase ultra-performance liquid chromatography-electrospray tandem mass spectrometry (**UPLC-ESI-MS**; Acquity H class, Waters Corp.). One microliter was injected on a C18 column (HSS T3, 2.1 × 100 mm, 1.7 µm; Waters Corp.) with a temperature of 40°C and a flow rate of 0.5 mL/min. The mobile phase consisted of 0.1% formic acid in water. The ESI-MS analysis was performed with a single quadrupole mass spectrometer (SQ Detector 2, Waters Corp.) operated in ES+ mode. The source temperature was 150°C with a desolvation temperature of 650°C and nitrogen gas flow of 1,200 L/h. Capillary and cone voltages were 0.50 and 38 V, respectively. Furosine was quantified using the charged ion 255.06 in selective ion monitoring mode. A 5-point standard curve was constructed, ranging from 15 to 150 µg/L, for quantification.

Sensory testing was conducted in accordance with the North Carolina State University Institutional Review Board for the Protection of Human Subjects in Research regulations. A trained sensory panel documented sensory attributes of milks on the day of manufacture. Each panelist (3 women, 3 men, ages 21 to 55 yr) had a minimum of 100 h of prior experience evaluating the sensory properties of fluid milk using the Spectrum method and an established lexicon for fluid milk (Meilgaard et al., 2007; Lee et al., 2017). Milks (30 mL) were dispensed into lidded 59-mL soufflé cups (Dart Container Corp., Mason, MI) with random 3-digit blinding codes. Samples were prepared with overhead lights off to prevent light oxidation. Milks were tempered to 10°C and evaluated using paper ballots. Each panelist evaluated each milk in duplicate.

Volatile compound analysis was evaluated from DSI and NVC milks as well as from the solid-phase microextraction (**SPME**) fibers collected during milk processing in the vacuum chamber. Volatile compounds were extracted from liquids using sorptive stir bar extraction (Prieto et al., 2010) with GC-MS and by SPME with GC-MS/MS. Milks were extracted in triplicate using the following methods adapted from Park and Drake (2016). Before analysis, the stir bars and thermal desorption unit tubes were conditioned for 1 h at 300°C. First, 5 mL of milk was added to a 10-mL glass vial. Next, 20 μL of internal standard was added (81 mg/kg of 2-methyl-3-heptanone in methanol, Sigma Aldrich). Sequential stir bar extraction was used because the adsorption of compounds onto the polydimethylsiloxane (**PDMS**) layer is affected by the addition of salt. Polar compounds are extracted more efficiently with salt, whereas hydrophobic compounds are extracted more efficiently without salt (Prieto et al., 2010). One PDMS-coated stir bar (10 mm × 0.5 mm thickness, Gerstel Inc.) was placed in the vial, sealed, and stirred for 1 h at 1,000 rpm. After 1 h, the stir bar was briefly rinsed in HPLC-grade water, dried, and placed in an autosampler tube (Gerstel Inc.), and 1 g of NaCl was added along with another PDMS stir bar into the sample vial. This stir bar was stirred for 1 h at 1,000 rpm, rinsed, dried, and placed in the same thermal desorption unit tube previously mentioned. Stir bars were injected using an autosampler (MPS Autosampler, Gerstel Inc.). The stir bars were desorbed at 250°C for 10 min (thermal desorption unit, Gerstel Inc.) and the volatile compounds were cryogenically trapped at −120°C (CIS 4, Gerstel Inc.). Volatile compounds were analyzed by GC-MS. An Agilent 7890B GC (Agilent Technologies Inc.) with an inert mass selective detector (model 5970A, Agilent Technologies Inc.) with a ZB-5 MS column (30 m × 0.25 mm × 0.25 μm; Phenomenex) was used to identify and quantify volatile compounds of interest (Park and Drake, 2016; Jo et al., 2018). Initial GC oven conditions were 40°C for 3 min with ramp rates of 10°C/min to 90°C, 5°C/min to 200°C and held for 10 min, and 20°C/min to 250°C and held for 5 min. Purge time was set to 1.2 min using helium as the carrier gas at a constant flow rate of 1 mL/min. Compounds were identified by comparison with the 2014 NIST mass spectral library (NIST, 2014), retention index, and retention time of authentic standards injected under identical conditions. Relative abundance of selected compounds was calculated using recovery of the internal standard.

Relative abundances of selected sulfur volatile compounds in milks were extracted by SPME and evaluated on an Agilent 7890B GC applied to an Agilent 7000C triple quad MS (MS/MS) and sulfur-selective flame photometric detector (**FPD**; Agilent Technologies Inc.) equipped with a ZB-5 MS (30 m length × 0.25 mm i.d. × 0.25 µm film thickness; Phenomenex) column. Five milliliters of milk and 20 µL of internal standard (2-methyl-3-heptanone in ethyl ether at 81 mg/kg) were added to 20-mL SPME autosampler vials with steel screw tops containing a silicone septum faced in Teflon (Microliter Analytical) in triplicate. Sample introduction was accomplished using a CTC Analytics CombiPal Autosampler (CTC Analytics). Vials were equilibrated for 25 min at 35°C with 4 s of pulsed 250 rpm agitation. A single 50/30 μm DVB/CAR/PDMS (Supelco) 1-cm fiber was used for all analyses. The SPME fiber was exposed to the samples for 40 min at a depth of 31 mm. The fiber was retracted and injected at 50 mm in the GC inlet for 5 min. The GC oven was initially held at 35°C for 3 min with a gradual increase of 10°C/min to 150°C, held for 1 min, and then increased at a rate of 20°C/min to 250°C and maintained for 5 min. The MS transfer line was maintained at 250°C with the quad at 150°C and source at 250°C. The flow rate of helium quench gas and nitrogen collision gas was 1.0 mL/min and 2.5 mL/min, respectively. Effluent from the capillary column was split 1:1 between the MS and FPD. The FPD for sulfur compounds was set to 325°C for transfer line, air 100 mL/min, and hydrogen 60 mL/min. Compounds were identified by comparison with the 2014 NIST mass spectral library (NIST, 2014), retention index, and retention time of authentic standards injected under identical conditions. Relative abundance of selected compounds was calculated using recovery of the internal standard.

Volatile compounds that were extracted in the vacuum chamber by SPME fibers during milk processing were identified qualitatively by GC-olfactometry (**GC-O**) as well as by GC-MS (described previously). The GC-O analysis was performed with a gas chromatograph with a flame-ionization detector and olfactometry port (model 6850, Agilent Technologies Inc.) and the same nonpolar column as stated above. Three highly trained sniffers (>50 h experience each) sniffed each sample once per replication and recorded aroma-active events. Oven temperature program was as follows: 40°C held for 3 min, 10°C/min gradual increase to 150°C, and 30°C/min gradual increase to 200°C held for 10 min. The GC-MS analysis was performed on an Agilent 7820A GC (Agilent Technologies Inc.) with an inert mass selective detector (model 5975 MSD, Agilent Technologies Inc.). Fibers were manually injected for GC-O. Column and oven temperature program were the same as above. All fibers were exposed in the column inlet at 250°C. Helium carrier gas was used on both GC-O and GC-MS at a rate of 1 mL/min with a purge time of 1.5 min.

Furosine, an indicator of heat treatment, was measured to ensure that the 2 milks received similar heat treatments and that flavor differences were not attributed to difference in heat load. Both milks had similar furosine values (55.37 vs. 52.94 mg of FUR/100 g of protein; *P* > 0.05). Flavor differences measured between NVC and DSI milks were therefore attributed to the effect of the vacuum cooler rather than a difference in heat treatment. The DSI milk was lower in sweet aromatic and cooked milky flavors by descriptive analysis compared with the NVC milk (*P* < 0.05; Table 1). Aroma intensity, viscosity, and astringency intensities were not different between the 2 milks (*P* > 0.05). These results are consistent with the hypothesis that DSI milk is lower in sweet aromatic flavor because of compounds being stripped from the milk by the vacuum cooler. Sulfur/eggy flavor was higher in DSI milk than in NVC milk (*P* < 0.05).

**Table 1:** Descriptive sensory means for DSI and NVC milks diluted to 8.6% solids^1^

Attribute	Process^2^	
	DSI	NVC
Aroma intensity	4.0^a^	3.3^a^
Sweet aromatic	0.7^b^	2.1^a^
Cooked milky	4.3^b^	4.6^a^
Sulfur/eggy	2.7^a^	1.8^b^
Viscosity	1.9^a^	1.9^a^
Astringency	2.3^a^	2.3^a^

a,b Means within a row without a common superscript are different (*P* < 0.05).

1 Means from descriptive analysis of 3 experimental replications. Sensory attributes were scored on a 0 to 15 scale; most fluid milk flavors fall between 0 and 5 on this scale (Lee et al., 2017).

2 DSI = ultrapasteurization via direct steam injection and vacuum cooling; NVC = ultrapasteurization via direct steam injection but without vacuum cooling.

Analysis of the SPME fibers exposed to the exit of the vacuum chamber showed that many odor-active compounds were removed during the UP process (Table 2). Of the compounds removed by vacuum cooling, important flavor volatiles can be organized into 3 categories: sulfur/eggy, cooked, and sweet aromatic volatiles. Hydrogen sulfide, dimethyl sulfide, and dimethyl disulfide have sulfur/eggy aromas (Jo et al., 2018) and were detected by GC-MS/MS and GC-O. Cooked compounds such as carbon disulfide and 2-methylbutanal (Jo et al., 2018) were also detected by SPME in the vacuum chamber. Sweet aromatic compounds—furaneol, maltol, δ-decalactone, and diacetyl (Karagül-Yüceer et al., 2001)—were also detected. These results suggest that beyond the direct measurement of the sensory and volatile profiles of the 2 liquid samples, we can qualitatively see that many important milk flavor compounds were removed by vacuum cooling. Analysis of GC data of the NVC and DSI milks further revealed the effect of vacuum cooling on milk flavor (Table 3). The DSI milk was lower in the sweet aromatic flavor compounds 2-heptanone, 2-nonanone, octanoic acid, and dodecanoic acid than NVC milk (*P* < 0.05, Table 3). The DSI milk was also lower (*P* < 0.05) in hydrogen sulfide, dimethyl sulfide, and carbon disulfide than the NVC milk. Extraction of volatile compounds from milks, similar to the qualitative SPME GC-O of vacuum chamber vapor, demonstrated that vacuum cooling removed aroma-active compounds.

**Table 2:** Aroma-active volatile compounds identified in the vacuum chamber outlet during direct steam injection–ultrapasteurization of skim milk by solid-phase microextraction (SPME) gas chromatography-olfactometry (GC-O)^1^

Compound	Identification	Odor description	RI
Hydrogen sulfide	RI, MS	Sulfur/egg	544
Dimethyl sulfide	RI, MS, O	Chemical/sulfur	564
Carbon disulfide	RI, MS	Cooked	568
2-Butanone	RI, MS	Plastic	596
Diacetyl	RI, MS, O	Diacetyl/buttery	607
2-Methylbutanal	RI, MS	Cooked/malty	645
3-Methylbutanal	RI, MS	Cooked/malty	665
Acetoin	RI, MS, O	Sweet/milky	701
Dimethyl disulfide	RI, MS, O	Earthy/sulfur	752
Dimethyl sulfoxide	RI, MS	Sulfur/garlic	767
Hexanal	RI, MS, O	Grass	808
Furfural	RI, MS, O	Barny/brothy	857
2-Heptanone	RI, MS	Cooked/milky	897
Heptanal	RI, MS	Earthy/fatty	898
Methional	RI, MS, O	Potato/earthy	920
Benzaldehyde	RI, MS	Cooked/nutty	944
Dimethyl trisulfide	RI, MS, O	Garlic/cabbage	984
1-Octen-3-one	RI, MS	Mushroom	990
Octanal	RI, MS	Grass	995
1-Octen-3-ol	RI, MS	Mushroom	1,003
Furaneol	RI, O	Sweet	1,061
Guaiacol	RI, O	Earthy	1,091
Maltol	RI, MS, O	Sweet	1,110
Sotolon	RI, MS	Cooked/spicy	1,120
2-Aminoacetophenone	RI, O	Tortilla/grain	1,354
γ-Decalactone	RI, MS	Sweet/caramel	1,486
δ-Decalactone	RI, MS, O	Fruity	1,494
δ-Undecalactone	RI, MS	Butter sweet	1,553
Dodecanoic acid	RI, MS	Soapy	1,593
γ-Dodecalactone	RI, MS	Sweet/grain	1,649
δ-Dodecalactone	RI, MS, O	Sweet/grain	1,779

1 RI = retention index (on ZB-5 column; Phenomenex); O = odor; MS = mass spectra.

**Table 3:** Relative abundance (μg/kg) of selected volatile compounds of direct steam injection–ultrapasteurized skim milk with (DSI) and without vacuum cooling (not vacuum cooled, NVC) diluted to the same solids content (8.6% wt/vol)^1^

Method and volatile compound^2^	NVC	DSI	*P*-value
SBSE-GC-MS			
1-Octanol	0.482^a^	0.381^a^	0.315
Decanal	3.33^a^	2.72^a^	0.472
Dodecanal	1.38^a^	1.40^a^	0.975
Hexanal	1.09^a^	1.11^a^	0.820
Heptanal	0.675^a^	0.618^a^	0.715
Nonanal	8.02^a^	6.59^a^	0.453
Octanal	0.451^a^	0.347^a^	0.216
E-2-octanal	0.0635^a^	0.0640^a^	0.990
E-2-hexenal	0.443^a^	0.924^a^	0.511
Undecanal	0.596^a^	0.467^a^	0.618
Furfural	0.509^a^	0.324^a^	0.268
2-Heptanone	1.35^a^	0.630^b^	0.028
2-Nonanone	0.719^a^	0.24^b^	0.008
δ-Decalactone	0.218^a^	0.161^a^	0.447
δ-Dodecalactone	0.167^a^	0.205^a^	0.320
γ-Dodecalactone	0.432^a^	0.260^a^	0.350
γ-Decalactone	0.401^a^	0.320^a^	0.674
δ-Nonalactone	0.468^a^	0.540^a^	0.150
Benzothiazole	0.924^a^	0.837^a^	0.786
Hexanoic acid	2.35^a^	1.99^a^	0.567
Heptanoic acid	0.554^a^	0.739^a^	0.406
Octanoic acid	8.01^a^	6.08^b^	0.077
Dodecanoic acid	4.30^a^	2.96^b^	0.035
SPME Triple Quad GC-MS/MS			
Hydrogen sulfide	15.5^a^	9.66^b^	0.002
Dimethyl sulfide	7.40^a^	1.85^b^	0.036
Carbon disulfide	5.42^a^	2.85^b^	0.047
Dimethyl disulfide	0.385^a^	0.076^b^	0.029
Dimethyl trisulfide	0.142^a^	0.085^b^	0.029
3-Methyl butanal	1.3^a^	0.935^b^	0.048
2-Methyl butanal	2.19^a^	1.14^b^	0.008
Benzaldehyde	1.225^a^	0.587^b^	0.026
Furaneol	0.489^a^	0.434^a^	0.094
Maltol	0.106^a^	0.106^a^	0.073
Diacetyl	0.515^a^	0.420^b^	0.011
1-Octen-3-one	1.40^a^	0.344^b^	0.038

a,b Means in the same column not sharing a common superscript are different (*P* < 0.05).

1 Each mean represents triplicate evaluations from 3 experimental replications.

2 SBSE-GC-MS = stir bar sorptive extraction GC-MS; SPME Triple Quad GC-MS/MS = solid-phase microextraction triple quadrupole GC-tandem MS.

The DSI milk was higher in sulfur/eggy flavor than the NVC milk (Table 1). This result seems to contradict the results obtained from GC, which showed that DSI milk was lower in sulfur/eggy volatile compounds than NVC milk. This discrepancy may be due to the complexity of the sensory perception of flavor. Another possible explanation is that during the DSI process, air may be incorporated, which can lead to oxidation of sulfur/eggy compounds. The presence of other compounds can modulate and change the detection thresholds of compounds (Green et al., 2010). Suppression of sulfur flavors may occur due to the presence of other background flavor compounds. After removal of other background flavor compounds by the vacuum chamber, sulfur/eggy flavor increases even though sulfur/eggy flavor compounds were decreased by vacuum cooling.

The flavor of UP milk varies with the methods used to pasteurize the milk (Lee et al., 2017). Flavor changes that occur during IND-UP have been associated with Maillard reactions and include sulfurous, cooked cabbage, and caramelized notes (Colahan-Sederstrom and Peterson, 2005; Potineni and Peterson, 2005; Kokkinidou and Peterson, 2014). Recently, Jo et al. (2018) confirmed that DSI-UP milk was higher in the sulfur compounds hydrogen sulfide, dimethyl trisulfide, and methional compared with IND-UP milk. Our research also suggests that these sulfur compounds may be more influential on milk flavor partly because of the effect the vacuum cooler has on these volatile compounds. The vacuum cooler caused distinct flavor differences between DSI and NVC milks. Analysis of the exiting vapor showed that many important milk flavor compounds were removed by the vacuum cooler.

Vacuum cooling of milk during UP removes aroma-active compounds and changes the flavor profile of milk. Sweet aromatic, sulfur/eggy, and cooked flavors are removed as measured by analysis of both the milk and the vapor removed by vacuum. The resulting DSI-UP milk has decreased sweet aromatic flavor and higher sulfur/eggy flavor. Flavor removal by DSI-UP becomes a further problem with highly flavored extended shelf life beverages. Flavors for beverages make up a large percentage of the formulation costs. Losing costly flavor compounds in the form of a vapor through the vacuum chamber would be less than ideal. Vacuum chamber coolers can be removed from the process and exchanged with a different type of cooler; however, dilution of the product will result. Therefore, this approach cannot be used for standard-of-identity dairy products but could be done for a formulated beverage that is batched at higher solids to meet the target solids with dilution of steam. Although DSI allows for a lower heat load on milk, the effect of the vacuum cooler may reduce desirable milk flavors and highlight less desired flavors.

## References

[bib1] Colahan-Sederstrom P.M., Peterson D.G. (2005). Inhibition of key aroma compound generated during ultrahigh-temperature processing of bovine milk via epicatechin addition. J. Agric. Food Chem..

[bib2] Datta N., Elliott A.J., Perkins M.L., Deeth H.C. (2002). Ultra high temperature treatment of milk: Comparison of direct and indirect modes of heating. Aust. J. Dairy Technol..

[bib3] Green B.G., Lim J., Osterhoff F., Blacher K., Nachtigal D. (2010). Taste mixture interactions: Suppression, additivity, and the predominance of sweetness. Physiol. Behav..

[bib4] Jo Y., Benoist D.M., Barbano D.M., Drake M.A. (2018). Flavor and flavor chemistry differences among milks processed by high-temperature, short-time pasteurization or ultra-pasteurization. J. Dairy Sci..

[bib5] Karagül-Yüceer Y., Drake M.A., Cadwallader K.R. (2001). Aroma-active components of nonfat dry milk. J. Agric. Food Chem..

[bib6] Kokkinidou S., Peterson D.G. (2014). Control of Maillard-type off-flavor development in ultrahigh temperature-processed bovine milk by phenolic chemistry. J. Agric. Food Chem..

[bib7] Lee A.P., Barbano D.M., Drake M.A. (2017). The influence of ultra-pasteurization by indirect heating versus direct steam injection on skim and 2% fat milks. J. Dairy Sci..

[bib8] Meilgaard M.C., Civille G.V., Carr B.T. (2007). Sensory Evaluation Techniques.

[bib9] NIST (National Institute of Standards and Technology) (2014).

[bib10] Park C.W., Drake M.A. (2016). Condensed milk storage and evaporation affect the flavor of nonfat dry milk. J. Dairy Sci..

[bib11] Potineni R.V., Peterson D.G. (2005). Influence of thermal processing conditions on flavor stability in fluid milk: Benzaldehyde. J. Dairy Sci..

[bib12] Prieto A., Basauri O., Rodil R., Usobiaga A., Fernandez L.A., Etxebarria N., Zuloaga O. (2010). Stir-bar sorptive extraction: A view on method optimization, novel applications, limitations and potential solutions. J. Chromatogr. A.

[bib13] Resmini P., Pellegrino L., Battelli G. (1990). Accurate quantification of furosine in milk and dairy products by a direct HPLC method. Ital. J. Food Sci..

